# The Role of Age, Religion/Spirituality, and Humor in the Optimism of Community-Dwelling Older Adults

**DOI:** 10.1093/geroni/igab046.3281

**Published:** 2021-12-17

**Authors:** Bonnie Kenaley, Eun hae Kim, Kimberly McClive-Reed, Zvi Gellis

**Affiliations:** 1 Boise State University, Boise, Idaho, United States; 2 Texas State University, San Marcos, Texas, United States; 3 University of Pennsylvania, NISKAYUNA, New York, United States; 4 University of Pennsylvania, Philadelphia, Pennsylvania, United States

## Abstract

Current literature reveals that perceived optimism decreases with age (D’Argembeau et al., 2011; Newby-Clark & Ross, 2003). However, replication of these studies is limited. In particular, a lack of investigations exists in examining optimism as individuals transition across older adulthood. Considering the dearth of literature that examines the influence of religion/spirituality and humor on the optimism of older adults, 203 members of Osher Lifelong Learning Institutes, age 65 years and older, from Idaho and California completed pen and paper or electronic surveys. The study used hierarchical multiple regression analysis to examine the impact of age, positive religion/spirituality coping skills, and humor on the optimism of community-dwelling older adults. The participant’s gender significantly explained a 19.3% variance in the optimism scores, whereas age did not significantly contribute to the model (R Change =.000). Positive religious or spiritual coping skills and humor significantly contributed to the variance in optimism scores, explaining a 2.3% and 21.6% variance, respectively. In the final model (F (1, 202, = 13.78, p = .000), all variables except age significantly contributed to the model with humor revealing the highest beta value (beta = .467, p = .000). The findings suggest that optimism is perceived differently by gender but does not change with age. While positive religious coping skills influence optimism, internal or external humor strategies may be more helpful to instill, promote or maintain optimism in older adults. The addition of humor assessment items in wellness evaluations and humor-infused interventions may foster optimism in community-dwelling older adults.

